# Intercostal Neuralgia in Women

**Published:** 1874-04-15

**Authors:** J. Milner Fothergill


### INTERCOSTAL NEURALGIA IN WOMEN.

By J. Milner Fothergill, M.D., M.R.C.P.

*From Obstet. Jour. of Great Britain and Ireland.*

THERE is no more marked form of disease than this particular form of neuralgia. It is commonly met with among the out-patients of every medical charity, and even in private practice. Indeed, it is the commonest affection met with among women of that class where neuralgia, unconnected with diathesis, might fairly be expected, viz.: among those where nutrition is defective, an essential in the production of neuralgia. It belongs to the reproductive period of woman's existence, and is but comparatively rarely seen after that time, and never, in my experience, before it. It is a troublesome and intractable malady unless approached vigorously and with relation to those disturbances of the reproductive organs with which it is so intimately associated. In almost every instance, leucorrhœa is present; usually either with amenorrhœa or menorrhagia; and in those cases which are not accompanied by leucorrhœa, the woman is usually suckling.

The pain is truly neuralgic; that is, according to Anstie, it comes in recurrent waves, or gusts, and is one-sided. I have never seen a case of this form of neuralgia where the pain was on both sides, and but rarely where it was on the right side. It is a left-side pain essentially. It is commonly called "pain in the side," and its truly neuralgic character is overlooked. A patient suffering from this affection gives a history to the following effect: She is weak and feeble, with black spots before her eyes, and has pain in her side and betwixt her shoulders, and very commonly dyspepsia, or constipation. In addition to this, she admits, more or less reluctantly, that she is much troubled with leucorrhœa, and usually has some uterine derangement. In the cases where this is not the case, she is suckling. In appearance, she usually presents a debilitated aspect, and very commonly is a dark and sallow woman, of lymphatic temperament; but by no means necessarily so; and women of a totally different character are found as sufferers from this feminine scourge. The tongue is usually clean, bright, and often silvery, without change of size, except in advanced or aggravated cases, when it is swollen and indented by the pressure of the teeth. She complains of pain in the side and betwixt the shoulders, and the painful spots are very tender upon pressure. In reality, these are the tender spots of Valleix; and one is found over or near the left apex, and the other at the posterior spinal rootlet of the nerve. The nerve usually affected is the sixth intercostal. Such is the malady in its ordinary aspect; and its features are singularly unvarying, so much so, indeed, that when "pain in the side" is complained of, the symptoms can be rapidly run up, often much to the patient's astonishment. This is especially the case as to to the uterine connections, which are often carefully concealed, and only admitted when the question is pressed.

As a rule, it may be said these cases are found among the married, and among servants who work hard and take little care of themselves; indeed, they often scarcely know how if they had the time to do so. In rare cases, women past the meno-pause have this ailment, commonly with its ordinary accompaniment leucorrhœa; at other times without it. It is a disease of debility whenever met, and is free from any association with those affections, syphilis and malaria, so productive of neuralgia. At times it is found in girls who are decidedly anæmic, and verging upon chlorosis; and tedious and ineffective is the treatment where the relations and concomitants of the neuralgia are overlooked, either from ignorance or carelessness.

The prognosis of the disease, like that of neuralgia generally, is good; but the progress is much affected by the treatment, and that again depends much on the knowledge of the ailment possessed by the medical adviser.

*Treatment.* — This must be conducted partly on general principles, partly in reference to the special indications. As to the first, we must remember the other two characteristics of genuine neuralgia given by Anstie, viz.: that it is aggravated by all depressing agents, and by increasing debility; and also that it is relieved by general improvement of the condition, and by the agents which tend to induce the latter change. My usual rule has been to give a combination of stimulants and tonics, and specially carbonate of ammonia with the ammonio-citrate of iron in an infusion of quassia. In a little time this may be advantageously changed for sulphate of quinine, muriate of iron and quassia. Recently, however, I have accompanied my friend Professor Ferrier to the West London Hospital, and compared notes with him. His favorite treatment is, to give the well-known mixture of gentian and rhubarb. In many cases, where the gastric symptoms are marked, this plan is unquestionably successful; but in others, the plan adopted by myself is more effective. The change, however, is almost certainly effective. In addition to this exhibition of internal remedies, belladonna plasters and the local application of mustard have been tried; but of course it is difficult to say with what effect, as other measures were combined with them.

The absolutely necessary part of the treatment is the attention to the local discharge. Whether this discharge is vaginal or uterine, I do not know, not having investigated the point. The use of the cold hip-bath, or where this is impracticable, or is badly borne, cold water bathings of the parts night and morning are necessary. To this may be added, in more obstinate cases, injections, either of cold water or the ordinary astringent mixtures. Without this local treatment is properly followed out, the progress of the case will be uncertain and disappointing.

Where there is menorrhagia, the usual plans of treatment of that affection may be blended with the measures given above. The remedies indicated in these cases are, however, rather of an astringent nature, their constipating effects being obviated by the administration of laxatives. In all cases, indeed, the bowels should be attended to; and for this purpose, aloes are well suited from their action on the hæmorrhoidal vessels. The action of the skirts hanging from the waist and squeezing the contents of the abdomen into the pelvis should not be forgotten; and everything calculated to produce pelvic congestion should be avoided.

Where the affection is associated with suckling, the child should be weaned forthwith, or, at least, the breast should be reserved for the night.

